# Why Are tRNAs Overproduced in the Absence of Maf1, a Negative Regulator of RNAP III, Not Fully Functional?

**DOI:** 10.1371/journal.pgen.1005743

**Published:** 2015-12-31

**Authors:** Magdalena Boguta

**Affiliations:** Department of Genetics, Institute of Biochemistry and Biophysics, Warsaw, Poland; Ohio State University, UNITED STATES

tRNA biosynthesis in the eukaryotic cell is a multistep pathway, involving transcription, 5' and 3' end maturation, intron removal, and numerous modifications of nucleotides. Most of the genes coding for each of the elements essential for tRNA biosynthetic activities were primarily identified by genetic selection in yeast [1]. In these studies, the parental strain contained a tRNA gene that had been converted to a nonsense suppressor. Given the appropriate genetic background, phenotypic loss of suppression was used to select mutants producing non-functional tRNA. Among other proteins controlling tRNA biosynthesis, this approach led to identification of Maf1, a global repressor of tRNA transcription that is activated in response to stress. The *maf1-1* mutant was originally selected in a genetic screen for decreased efficiency of tRNA suppressor *SUP11* (tRNA Tyr/UAA) in budding yeast, *Saccharomyces cerevisiae* [2]. The role of Maf1 has been suggested by tRNA accumulation in *maf1∆* cells, observed regardless of the repressive growth conditions [3]. An analogous decrease of tRNA-suppressor (tRNA Ser/UCA) activity was detected for the *maf1∆* mutant in *Schizosaccharomyces pombe* [4]. Interestingly, the effect of Maf1 on the efficiency of tRNA-mediated suppression is contrary to that expected. Although one would assume that increased cellular tRNA levels should improve the efficiency of tRNA-mediated nonsense suppression, data show the opposite is true.

Despite nearly two decades since the original discovery, the mechanism by which tRNA accumulation in the *maf1∆* mutant leads to the antisuppressor phenotype is still not understood. The simplest hypothesis is that tRNAs transcribed in *maf1∆* cells are incompletely processed, hypomodified, or fail to be appropriately delivered to ribosomes. It is worth noting that both primary transcripts and end-processed, intron-containing tRNA precursors were abnormally abundant in the absence of Maf1, and the nuclear export machinery was overloaded [5]. It was, however, unknown which processes in the tRNA maturation pathway were saturated by the increased amounts of primary transcripts in cells lacking Maf1.

**Fig 1:**
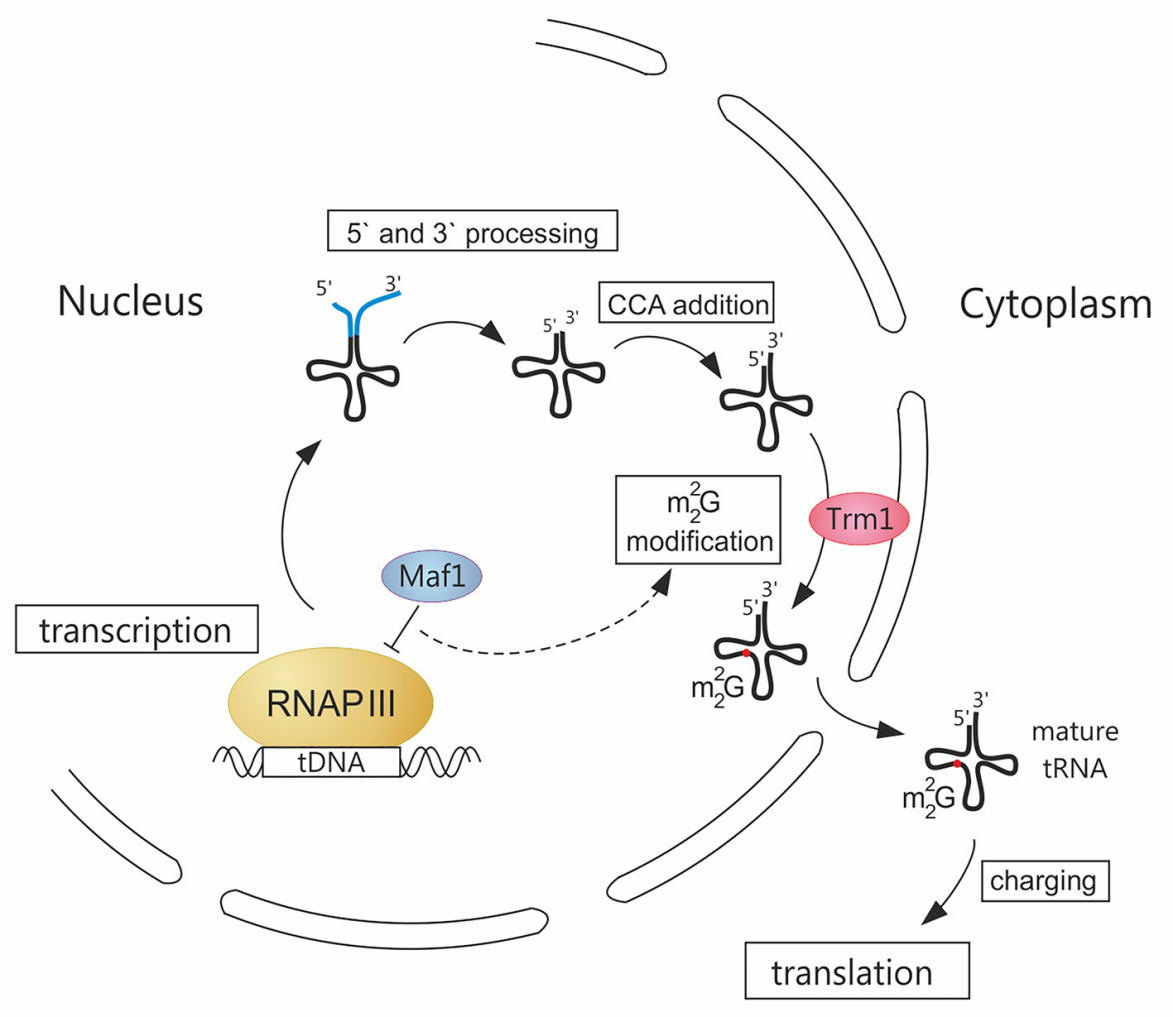
A novel link between RNAP III transcription and tRNA modification. Transcription of tRNA precursors by RNA polymerase III (RNAP III) in the nucleus is controlled by the general repressor Maf1. Initial 5' and 3' end maturation including addition of CCA sequence, takes place in the nucleus. Following m^2^_2_G26 modification by Trm1 methyltransferase tethered to the inner nuclear membrane, tRNAs are exported to the cytoplasm, charged, and directed to translation machinery. A previously unknown link connecting RNAP III activity and m^2^_2_G26 efficiency, due to a limiting amount of Trm1, is designated by a dashed line. Splicing of intron-containing tRNA precursors and other modifications that can be added in each step of tRNA biosynthesis are not presented on the scheme.

The current study by Arimbasseri and colleagues [4] solves a long-term conundrum: why tRNAs overproduced in the absence of Maf1 are not fully functional. Their elegant work makes a convincing case for the saturation of the dimethyltransferase Trm1 playing a crucial role in the mechanism by which Maf1 affects tRNA suppression. By using tRNA-HydroSeq technique to examine tRNA modification levels in *S*. *pombe* on the global scale, the authors have shown that Trm1 substrates are not fully modified even in wild type cells. Further decrease of Trm1-mediated G26 dimethylation on certain tRNAs was detected in the *maf1∆* mutant. Consequently, hypomodification of G26 due to limited Trm1 reduces the activity of tRNA-suppressor-Ser/UCA and accounts for antisuppression. This hypothesis was validated by genetic complementation of the antisuppressor phenotype of the *maf1∆* mutant in fission yeast by overproduced Trm1 [4]. Moreover, treatment with rapamycin or overexpression of Maf1 reduced tRNA transcription with increase in the m^2^_2_G26 content of tRNA-suppressor-Ser/UCA and its specific activity for suppression. Additionally, RNA polymerase III (RNAP III) mutations associated with hypomyelinating leukodystrophy decreased tRNA transcription, increased m^2^_2_G26 efficiency, and reversed antisuppression. Taken together, these results demonstrate that increases or decreases in global RNAP III activity lead to inverse changes in the efficiency of m^2^_2_G26 modification of specific tRNAs. Thus, a previously unknown link connecting RNAP III activity and m^2^_2_G26 efficiency is due to a limiting amount of Trm1 (Fig 1). This link has been conserved through evolution, since the authors showed that the increase of m^2^_2_G26 content in specific tRNAs in response to starvation was detected in the human embryonic kidney. Moreover, increased production of Trm1 in *S*. *cerevisiae maf1∆* cells led to reversal of the antisuppression phenotype, as was also observed in fission yeast. In both these cases, however, reversal of antisuppression by overproduced Trm1 is incomplete, suggesting that other factors involved in tRNA maturation might also be saturated in the context of increased tRNA synthesis in *maf1*Δ [4].

Why might tRNA modification by Trm1 be limited? A simple explanation could be that the level of Trm1 protein is regulated by the growth conditions that affect tRNA transcription. This was, however, excluded experimentally, suggesting that Trm1 activity may be controlled by a posttranslational mechanism. Another interesting possibility is that Trm1 modification might be limited by tRNA retention time in the nucleus. In *S*. *cerevisiae*, Trm1 is tethered to the inner nuclear membrane via a specific amino acid sequence tract [6]. Although nuclear residence may limit the time during which a nascent pre-tRNA transcript might have access to acquire the m^2^_2_G26 modification, retrograde RNA transport should theoretically allow iterative access to Trm1 [7]. Therefore, the mechanism by which cells maintain Trm1 activity in a functionally limiting amount is unclear.

G26 resides at the junction between the D-stem and the anticodon stem, and its *N2*-dimethylation, which interferes with normal Watson-Crick base pairing, may contribute to prevention of tRNA misfolding. It is also notable that treatment of cells with 5-flurouracil (5FU), which is incorporated into RNA, sensitizes *S*. *cerevisiae* to loss of genes that encode tRNA modification enzymes whose nucleoside targets localize at or near the stems junction and include gene-encoding Trm1 [8]. These observations, together with evidence that m^2^_2_G26 can stabilize correctly folded anticodon stems [9], suggest that m^2^_2_G26 may enhance tRNA-specific activity by improving the fit in the ribosome.

Although such a speculative role of Trm1 is beyond the scope of the current study, there are examples of tRNA modification enzymes that affect posttranscriptional regulatory mechanisms. Trm9, a methyltransferase that catalyzes modification of wobble bases in the tRNA anticodon, enhances the translation of the class of transcripts overrepresented with specific arginine and glutamic acid codons, which encode key damage response proteins [10]. Next, modification of tRNA-Lys/UUU by an elongator is essential for efficient translation of stress mRNAs [11]. Finally, loss of tRNA anticodon wobble uridine modification slows translation at cognate codons, leading to widespread protein aggregation [12]. Because of its hierarchical substrate preference, convincingly documented by Arimbasseri and colleagues, Trm1 may also contribute to maintaining proteome integrity.
